# Cognitive performance of older adults with a low level of education with and without depression

**DOI:** 10.1590/1980-57642021dn15-010013

**Published:** 2021

**Authors:** Ana Julia de Lima Bomfim, Natália Mota de Souza Chagas, Lívio Rodrigues Leal, Rebeca Mendes de Paula Pessoa, Bianca Letícia Cavalmoretti Ferreira, Marcos Hortes Nisihara Chagas

**Affiliations:** 1 Department of Neurosciences and Behavioral Sciences, Universidade de São Paulo – Ribeirão Preto, SP, Brazil.; 2 Research Group on Mental Health, Cognition and Aging, Universidade Federal de São Carlos – São Carlos, SP, Brazil.; 3 Bairral Institute of Psychiatry – Itapira, SP, Brazil.

**Keywords:** aging, depression, cognition, mental health, educational status, envelhecimento, depressão, cognição, saúde mental, escolaridade

## Abstract

Major depression can develop in individuals aged 60 years or older and is commonly associated with cognitive decline in this population, especially the domains of working memory, attention, executive functions, and processing speed. Schooling is a protective factor with regard to cognitive decline.

**Objective:**

To compare the cognitive performance of community-dwelling older adults with a low level of schooling with and without major depression.

**Methods:**

A descriptive, analytical, cross-sectional study was conducted with 22 community-dwelling older adults with depression and 187 without depression. The following assessment tools were employed: Mini Mental Health Examination, Brief Cognitive Screening Battery, Consortium to Establish a Registry for Alzheimer’s Disease (CERAD), Digit Span Test (forward and backward), and an object similarity test.

**Results:**

No statistically significant differences were found between the groups with and without depression on any of the tests.

**Conclusions:**

This study demonstrated that there are no differences in the cognitive performance of older people with and without depression on neurocognitive tests commonly used in clinical practice. Future studies with different designs and methods as well as specific tests for older people with a low level of schooling could assist in the understanding of these relations and the mechanisms involved.

## RESUMO.

A depressão maior pode se manifestar em indivíduos com 60 anos ou mais e, comumente, está associada ao declínio cognitivo, especialmente nos domínios memória de trabalho, atenção, função executiva e velocidade de processamento. Nesse contexto, a escolaridade é um fator de proteção em relação ao declínio cognitivo.

**Objetivo:**

Comparar o desempenho cognitivo entre idosos de baixa escolaridade da comunidade com e sem depressão maior.

**Métodos:**

Trata-se de um estudo transversal, descritivo e analítico. Foram selecionados 22 idosos da comunidade com depressão e 187 idosos sem depressão, que foram avaliados por meio dos seguintes instrumentos: Mini-Exame do Estado Mental (MEEM), Bateria Breve de Rastreio Cognitivo (BBRC), *Consortium to Establish a Registry for Alzheimer’s Disease* (CERAD), teste de extensão de dígitos de ordem direta e inversa, e um teste de semelhança de objetos.

**Resultados:**

Não foram encontradas diferenças estatisticamente significativas entre os grupos com depressão e sem depressão em nenhum dos testes aplicados.

**Conclusões:**

O presente estudo demonstrou que não existem diferenças no desempenho cognitivo de idosos com e sem depressão em testes neurocognitivos comumente utilizados na prática clínica. Estudos futuros com métodos e delineamentos diferentes, com testes específicos para idosos com baixa escolaridade, podem auxiliar na compreensão dessas relações e dos mecanismos envolvidos.

## INTRODUCTION

Major depression can develop in individuals aged 60 years or older, among whom the prognosis is worse, the course of the disease is more persistent, and the relapse rate is higher in comparison to younger individuals; moreover, concomitant cognitive decline is also often found in this population.^1^ ^,^ ^2^ Approximately 30% of older adults with depression exhibit cognitive decline, especially in the domains of working memory, attention, executive functions, and processing speed.^2^ ^,^ ^3^

Concomitant depression and cognitive decline exert a negative impact on quality of life and functional capacity, along with an increase in the relapse rate of depression, a delayed response to treatment, and greater use of specialized services.^4^ Moreover, cognitive impairment can persist after effective treatment for depression.^5^ ^,^ ^6^ Approximately 45% of patients with previous depression continue to exhibit significant cognitive impairment even after the treatment and remission of depressive symptoms.^7^

Evidence indicates that a current or past history of depression is a risk factor for the decline in cognitive function, which, in turn, is a predictor of future depressive symptoms.^8^ Moreover, social isolation, a low level of schooling, a poor socioeconomic status, and the occurrence of cardiovascular disease, such as hypertension, are risk factors shared by both depression and cognitive decline.^9^ ^,^ ^10^

The prevalence of cognitive impairment is higher among individuals with a low level of schooling.^11^ In Brazil, the prevalence is 18.7% among older adults and both age group and a low level of schooling are directly associated with the increase in cognitive impairment in this population.^11^ Moreover, even with no evidence of cognitive decline based on clinical history and examinations, individuals with a low level of schooling can perform poorly on cognitive tests.^12^

Although diverse cognitive assessment tools have been developed to screen for the decline in cognitive functions, such as the Mini-Mental State Examination (MMSE) and Montreal Cognitive Assessment (MoCA), educational level has implications with regards to the skills that are assessed using these instruments.^13^ Therefore, the prevalence of cognitive decline may be overestimated, especially in low- and middle-income countries, such as Brazil, as a good performance on cognitive screening tests is influenced by schooling.^12^

Evidence points to a deficit in the communication of brain regions underlying the performance on cognitive tasks among older adults with a low educational level.^14^ ^,^ ^15^ Thus, older people with a higher level of schooling have a compensatory network and can employ different strategies when performing a cognitive task, indicating that a higher level of education can contribute to greater cognitive reserve.^16^

Like cognitive impairment, a low level of schooling can be considered a risk factor for depression and the interaction between the two exerts an influence on the performance of neuropsychological tests.^17^ Therefore, the aim of the present study was to compare the cognitive performance of community-dwelling older adults with low schooling with and without major depression using a cognitive assessment protocol that encompasses memory, language, executive functioning, abstraction, and attention.

## METHODS

### Setting and participants

This study was conducted in the city of São Carlos, located in the state of São Paulo, Brazil. The participants were selected from a study for the screening of psychiatric disorders in the coverage area of a family health unit (Brazilian primary care modality). All homes were visited and a total of 289 older adults were invited to participate. Five declined, two were bedridden, and 15 did not undergo the psychiatric interview. Thus, 267 older adults were submitted to a diagnostic psychiatric assessment.

The exclusion criteria were severe vision or hearing impairment that could affect the understanding of the questions and tests and a diagnosis of a major neurocognitive disorder, psychotic disorder, intellectual deficiency, bipolar affective disorder, schizophrenia, or epilepsy. Fourteen individuals with more than eight years of schooling (two from the group with depression and 12 from the group without depression) were also excluded. The final sample was composed of 209 older adults, who were divided into two groups: 1) those with major depression (n=22) and 2) those without major depression (n=187).

The group with depression had eight individuals with comorbid anxiety disorder [general anxiety (n=6), specific phobia (n=4), and social anxiety disorder (n=1)]. Only two individuals in the group used a therapeutic dose of antidepressant medication (fluoxetine 40 mg/day and escitalopram 10 mg/day)]. In the group without major depression, 68 individuals had anxiety disorder [general anxiety (n=37), specific phobia (n=25), and social anxiety disorder (n=6)]. Five individuals in this group took a therapeutic dose of antidepressant medications (escitalopram 10 mg/day, sertraline 50 mg/day, fluoxetine 20 mg/day, and nortriptyline 50 mg/day.

#### Procedures

For the diagnosis of psychiatric disorder, the participants were evaluated during a detailed clinical interview performed by three psychiatrists (NMSC, RMPP, and LRL) on the basis of the guidelines of the Diagnostic and Statistical Manual of Mental Disorders (DSM-5) published by the American Psychiatric Association,^18^ which has a structure for diagnostic investigations and offers screening questions for the disorders listed in the DSM-5.^19^

The participants were also evaluated using an assessment protocol that encompassed sociodemographic characteristics and a battery of cognitive tests, which was performed by five trained gerontologists. The evaluations were conducted in the homes of the participants with a maximum interval of 30 days between evaluations. The data were collected between March 2016 and February 2017. There was no specific order for these steps of the study.

All volunteers agreed to participate by signing a statement of informed consent, which had received approval from the Human Research Ethics Committee of the Federal University of São Carlos (certificate number: 48602515.5.0000.5504).

The cognitive assessment consisted of the following tools.

#### Mini Mental State Examination

The MMSE is commonly used to screen for cognitive decline. It was created by Folstein et al.^20^ and translated into Brazilian Portuguese by Bertolucci et al.^21^ The MMSE is composed of domains that enable an objective assessment of spatial and temporal orientation, registration, registration recall, attention, calculation, and language. The cutoff point for cognitive impairment differs depending on the degree of schooling. The total score ranges from 0 to 30 points, with lower scores denoting greater cognitive decline.^20^

#### Cognitive Screening Battery

The BCSB is used to identify individuals with dementia in epidemiological studies^22^ and employs figures for the assessment of memory. The battery consists of naming, incidental memory, immediate recall, learning, delayed recall, and recognition as well as a verbal fluency test and clock-drawing test. The BCSB has good accuracy in populations with high illiteracy rates or low levels of schooling.^23^

##### Similarity Subtest of the Cambridge Examination for Mental Disorders (CAMDEX)

The similarity subtest of the CAMDEX consists of four questions for assessing the capacity for abstraction.^24^ The evaluator states the name of two objects and the participant must say how the objects are similar. For example: “In what way are a shirt and dress similar?”. The score on this subtest ranges from 0 to 8 points, with a higher score denoting a better performance. This instrument has been translated and adapted to Portuguese.^25^

#### Digit span test (forward and backward) from the Wechsler Memory Scale revised

The digit span test is comprised of seven pairs of numeric sequences.^26^ ^,^ ^27^ The test is administered in forward and backward sequences. The sequences have three to nine numbers in the forward test and two to eight numbers in the backward test. The examiner reads the sequence of numbers and the respondent repeats them. The examiner reads the sequence with a one-second interval between numbers and the sequence must be repeated after immediately after the reading. The test ends after mistakes occur on two consecutive sequences.

#### Consortium to Establish a Registry for Alzheimer’s Disease

The CERAD is a battery of neuropsychological tests developed by Morris et al.^28^ to establish an assessment standard for Alzheimer’s disease. The battery is composed of the following cognitive tests: verbal fluency (animals), 15-item Boston naming test, word list memory test, constructive praxis, recognition list, and praxis recall.^29^

#### Data analysis

Descriptive analysis was performed to characterize the sociodemographic profile of the groups. The Kolmogorov-Smirnov test was used to determine the normality of the data. The Student’s t-test and Mann-Whitney test were used to evaluate differences between groups according to the distribution of the variables, and the chi-square test was used for the comparison of categorical variables. Statistical analysis was performed with the aid of the SPSS 23.0 program, with the level of significance set at 5% (p<0.05).

## RESULTS

The clinical-demographic data of the sample are displayed in Table 1. No significant differences between groups were found regarding age (p=0.739), sex (p=0.256), family income (p=0.635), or schooling (p=0.266). Mean schooling was low in both groups. A statistically significant difference was found for polypharmacy, as the use of five or more medications was more frequent in the group with depression (p=0.010). No significant differences between groups were found regarding self-reported heart disease, hypertension, diabetes, or being a smoker or ex-smoker.

**Table 1:** Clinical and demographic characteristics of the groups with and without depression.

		Major depression (n=22)	Without major depression (n=187)	p-value
		Mean (SD)		
Age (years)		71.40 (±9.63)	70.05 (±7.23)	0.739
Schooling (years)		2.22 (±2.06)	2.85 (±2.27)	0.266
Family income (minimum wage)		2.55 (±1.38)	2.64 (±1.23)	0.635
		n (%)		
Sex				
	Female	15 (68%)	109 (58%)	0.256
	Male	7 (32%)	78 (43%)	
Polypharmacy				
	Yes	12 (55%)	51 (27%)	0.010*
	No	10 (45%)	136 (73%)	
Diabetes				
	Yes	7 (32%)	49 (26%)	0.348
	No	15 (68%)	138 (74%)	
Arterial hypertension				
	Yes	16 (73%)	114 (61%)	0.162
	No	6 (27%)	73 (39%)	
Heart disease				
	Yes	5 (23%)	35 (19%)	0.363
	No	17 (77%)	152 (81%)	
Smoker/ex-smoker				
	Yes	7 (32%)	76 (41%)	0.309
	No	15 (68%)	111 (59%)	

SD: standard deviation;

* statistically significant result.

Table 2 displays the mean and standard deviation values according to the cognitive domains. No significant differences between groups were found for any of the cognitive domains evaluated.

**Table 2:** Mean and standard deviation (±) values according to the cognitive domains evaluated between groups with and without depression.

		Major depression (n=22)	Without major depression (n=187)	p-value
		Mean (SD)		
Global cognition				
	MMSE	23.31 (±3.88)	22.88 (±3.82)	0.489
Memory				
	BCSB – incidental memory, immediate recall and learning	21.89 (±5.50)	21.00 (±5.23)	0.560
	BCSB – delayed recall	7.42 (±1.95)	6.94 (±2.34)	0.817
	BCSB – recognition	8.26 (±2.80)	8.82 (±2.08)	0.200
	CERAD – word list	14.15 (±5.75)	12.64 (±5.46)	0.516
	CERAD – delayed recall	3.84 (±2.43)	3.15 (±2.42)	0.303
	CERAD – recognition list	7.31 (±2.88)	7.64 (±2.54)	0.541
Language				
	Boston naming test	11.00 (±3.41)	11.44 (±2.49)	0.442
	Verbal fluency test	10.84 (±2.75)	10.76 (±3.63)	0.778
Executive functions				
	Clock-drawing test	5.10 (±2.75)	4.96 (±3.52)	0.867
	CERAD – constructive praxis	5.26 (±3.21)	5.53 (±3.092)	0.812
	CAMDEX similarity subtest	3.15 (±2.56)	2.69 (±2.10)	0.319
Attention				
	Digit span test (forward)	4.42 (±1.21)	4.57 (±1.20)	0.606
	Digit span test (backward)	2.10 (±1.44)	2.37 (±1.16)	0.574

SD: standard deviation; MMSE: Mini Mental State Examination; BCSB: Brief Cognitive Screening Battery; CERAD: Consortium to Establish a Registry for Alzheimer’s Disease.

## DISCUSSION

In the present study, no significant differences were found between the groups with and without depression with regard to cognitive domains. This finding differs from data described in the majority of studies in the literature, as an association between depressive symptoms and cognitive impairment has been reported in both cross-sectional^4^ ^,^ ^5^ ^,^ ^30^ ^,^ ^31^ ^,^ ^32^ and longitudinal^33^ ^,^ ^34^ studies.

A cross-sectional study conducted by Giri et al.^30^ evaluated the association between cognitive decline and depressive symptoms in a sample of 538 older people using the MMSE and 30-item Geriatric Depression Scale (GDS-30). Approximately 51% of the sample had 11 or more years of schooling. On the basis of the findings, depression was considered a predictive factor for cognitive decline, and cognitive impairment was associated with an increased risk of depression.

Likewise, Bunce et al.^33^ evaluated the temporal association between depressive symptoms and cognitive function in 896 community-dwelling older people between 70 and 97 years of age for a period of four years. The participants were divided into two groups: those with up to two depressive symptoms and those with more than two depressive symptoms. Mean schooling was 11.39±2.64 and 11.28±2.45 years, respectively. The results indicated that the presence of depressive symptoms exerts an influence on processing speed and reaction time, suggesting that depression may occasionally precede cognitive decline. The studies cited above used the presence of depressive symptoms to evaluate the association with cognition, which can facilitate the findings. Moreover, it is not always possible to clinically differentiate depressive and cognitive symptoms, as concentration problems and difficulties making decisions are commonly found in depressive conditions.

Regarding studies conducted in Brazil, Novaretti and Nitrini^31^ evaluated the performance of older adults with depression using the BCSB and CERAD. The sample was composed of 25 individuals with late-onset depression (mean age: 73.6±6.6 years; mean schooling: 9.1±5.7 years) and 30 healthy individuals (mean age: 73.8±5.8 years; mean schooling: 9.1±5.4 years). The group with depression had a poorer performance on the CERAD in the domains of verbal fluency (animal category) and word list recall and on the BCSB in the domains of learning and verbal fluency (fruit category). However, the groups were not matched for sex and the schooling of the sample was higher than that in the present sample, which may explain the divergent results.

A cross-sectional study conducted in the city of São Paulo, Brazil, evaluated the association between cognitive performance and both sociodemographic and health-related variables in a sample of 384 older adults (65 years of age or older), among whom 79.6% had less than four years of schooling. Cognition was assessed using the MMSE and BCSB. The results showed that age, sex, schooling, and depressive symptoms exerted significant influences on the cognitive performance of the individuals evaluated.^32^ However, the coefficient of partial determination (partial R^2^) in this study was 0.018, indicating that, despite being statistically significant, the presence of depressive symptoms only explained 1.8% of the variation in the MMSE scores.

The descriptions offered above reveal divergences in the findings reported in the literature. In the present investigation, no significant differences were found between the two groups regarding any cognitive domain. A possible hypothesis for this finding regards the level of schooling in the sample, as most studies that found differences between older groups with and without depression were conducted in high-income countries with samples that had higher levels of schooling.^4^ ^,^ ^5^ ^,^ ^30^ ^,^ ^33^ ^,^ ^34^ Thus, the low level of schooling in both groups of the present study may have influenced the results, as most of the test scores were low. Therefore, the determination of differences between the groups may have been hindered due to the floor effect. which can hinder the detection of differences in comparative analysis, facilitating the occurrence of type II errors.

As educational level exerts a direct influence on the performance of cognitive assessment tools, the choice of the cognitive battery to be used should always take this aspect into consideration. Moreover, the interpretation of the results of cognitive tests administered to older adults with a low level of schooling is questionable, as it is not possible to differentiate (especially in studies with a cross-sectional design) whether the poor performance is due to the low level of schooling or cognitive decline. Bento-Torres et al.^35^ found a poorer cognitive performance among older adults with low schooling (1 to 7 years) compared to those with eight or more years of schooling. The older adults in this study did not have any previous or current history of traumatic brain/head trauma, stroke, language impairment, chronic alcoholism, neurological diseases, memory problems or depressive symptoms, and had normal scores on the MMSE.

Another important aspect is that most epidemiological studies use scales to evaluate the presence of depression or depressive symptoms. The gold standard for the diagnostic evaluation would be the use of a semi-structured clinical interview, as this strategy would diminish the possibility of diagnostic errors. For instance, the GDS, which is widely used, can have different cutoff points depending on clinical comorbidities,^36^ ^,^ ^37^ and the presence of a symptom does not necessarily indicate that it can be attributed to depression. The GDS itself has an item directly related to memory impairment.^38^

The present study had limitations that should be considered, such as the absence of a measure for quantifying depression and the non-use of cognitive tests specific for respondents with low levels of schooling and for the evaluation of other domains, such as executive functions, attention, and processing speed. Moreover, the occurrence of anxiety disorders in some of the participants may have exerted an influence on the results and should also be considered a limitation of this study.

On the basis of the present findings, the identification of cognitive impairment in older adults with depression and a low level of schooling may be more complex and it is essential for the assessment to be as thorough as possible, considering the characteristics of the population, the cognitive battery used and the instrument employed for the diagnosis of depression. Cognitive instruments that are able to detect subtle differences and that can be adjusted for low levels of schooling are preferable. In the evaluation of depression, the use of a structured or semi-structured clinical interview is ideal, preferably based on the criteria of the DSM. Moreover, the scarcity of studies addressing the impact of depression on the cognitive performance of older adults with low schooling underscores the need for further investigations with different methods.

In the present study, older adults with low schooling and with depression did not have a poorer performance in comparison to those without depression on cognitive tests evaluating general cognition, memory, language, executive functions, and attention. Future studies with different designs and methods as well as specific tests for older people with a low level of schooling could assist in the understanding of these relations and the mechanisms involved.
